# Anatomical targeting for electrode localization in subthalamic nucleus deep brain stimulation: A comparative study

**DOI:** 10.1111/jon.13133

**Published:** 2023-06-08

**Authors:** Thomas Tonroe, Hugh McDermott, Patrick Pearce, Nicola Acevedo, Wesley Thevathasan, San San Xu, Kristian Bulluss, Thushara Perera

**Affiliations:** ^1^ Bionics Institute East Melbourne Victoria Australia; ^2^ School of Engineering RMIT University Melbourne Victoria Australia; ^3^ DBS Technologies Pty Ltd East Melbourne Victoria Australia; ^4^ Medical Bionics Department The University of Melbourne East Melbourne Victoria Australia; ^5^ Department of Neurosurgery St Vincent's Hospital Melbourne Fitzroy Victoria Australia; ^6^ Centre for Mental Health Swinburne University of Technology Melbourne Victoria Australia; ^7^ Department of Neurology Austin Hospital Heidelberg Victoria Australia; ^8^ Department of Medicine The University of Melbourne Parkville Victoria Australia; ^9^ Department of Neurology The Royal Melbourne Hospital Parkville Victoria Australia; ^10^ Department of Neurosurgery Cabrini Hospital Malvern Victoria Australia; ^11^ Department of Neurosurgery Austin Hospital Heidelberg Victoria Australia; ^12^ Department of Surgery The University of Melbourne Parkville Victoria Australia

**Keywords:** anatomical targeting, automation, deep brain stimulation, electrode localization, neuroimaging, Parkinson's disease, subthalamic nucleus

## Abstract

**Background and Purpose:**

In deep brain stimulation (DBS), accurate electrode placement is essential for optimizing patient outcomes. Localizing electrodes enables insight into therapeutic outcomes and development of metrics for use in clinical trials. Methods of defining anatomical targets have been described with varying accuracy and objectivity. To assess variability in anatomical targeting, we compare four methods of defining an appropriate target for DBS of the subthalamic nucleus for Parkinson's disease.

**Methods:**

The methods compared are direct visualization, red nucleus‐based indirect targeting, mid‐commissural point‐based indirect targeting, and automated template‐based targeting. This study assessed 226 hemispheres in 113 DBS recipients (39 females, 73 males, 62.2 ± 7.7 years). We utilized the electrode placement error (the Euclidean distance between the defined target and closest DBS electrode) as a metric for comparative analysis. Pairwise differences in electrode placement error across the four methods were compared using the Kruskal‐Wallis *H*‐test and Wilcoxon signed‐rank tests.

**Results:**

Interquartile ranges of the differences in electrode placement error spanned 1.18‐1.56 mm. A Kruskal‐Wallis *H*‐test reported a statistically significant difference in the median of at least two groups (*H*(5) = 41.052, *p* < .001). Wilcoxon signed‐rank tests reported statistically significant difference in two comparisons: direct visualization versus red nucleus‐based indirect, and direct visualization versus automated template‐based methods (*T* < 9215, *p* < .001).

**Conclusions:**

All methods were similarly discordant in their relative accuracy, despite having significant technical differences in their application. The differing protocols and technical aspects of each method, however, have the implication that one may be more practical depending on the clinical or research application at hand.

## INTRODUCTION

Subthalamic nucleus deep brain stimulation (STN DBS) is an effective and widely utilized treatment for people living with Parkinson's disease (PD).^1^ The STN is a small (approximately 3 × 5 × 12 mm) structure within the basal ganglia.^2^ While the exact location of the ideal stimulation site within the STN is under debate, electrode placement within the dorsolateral (motor) region of the STN is well established.^3^ Accurate placement of electrodes within this functional subregion improves therapeutic effectiveness.^4^

Error in electrode placement by merely 2 mm can reduce efficacy and result in stimulation‐induced side effects.^5^ To confirm accurate placement, electrode localization is performed. Localizing electrodes determines electrode positions relative to anatomy, providing clinicians and researchers direct insight into therapeutic outcomes.^6^, ^7^ It also enables the development of metrics for use in studies assessing new DBS techniques. Localization is achieved by co‐registering pre‐ and postoperative imaging and is a prerequisite to quantifying how close implanted electrodes are to an anatomical target or “sweet spot.”^8^ The anatomical target used as a reference can be defined broadly in three ways—direct visualization, indirect methods, and template‐based methods.

Direct visualization involves the selection of anatomical target coordinates by expert clinicians on high‐resolution preoperative MRI.^9^ As the current clinical standard, direct visualization allows for individualized targeting of the STN.^10^ Yet it is subjective and resource intensive, requiring experienced clinicians to manually mark the target. This results in significant heterogeneity between experts in anatomical target placement within the dorsolateral STN.^1^ This variability in targeting introduces error and restricts multicenter collaboration when performing electrode localization analysis.

Indirect methods involve the usage of standardized offsets from neighboring brain structures to target the STN.^11^ Indirect methods were developed when imaging modalities of the time made visualization of the STN difficult (<3T MRI). These offsets correspond with an average target within the dorsolateral STN. The use of more readily identifiable structures than the STN and standardized offsets may reduce subjectivity in targeting the STN. Standardized offsets from the visually identified red nucleus (RN) and mid‐commissural point (MCP) have been reported.^12^, ^13^ However, markup of reference structures remains subjective, and interindividual variations in brain morphology might produce comparable errors to direct visualization.^11^

Modern template‐based methods utilize population‐based templates, created by merging MRI datasets into a single “average” brain volume.^14^, ^15^ Similarly, average targets for STN DBS are derived from population‐based studies of DBS outcomes and can be defined in the same standard coordinate system.^16^ These average targets can then be applied to an individual's imaging after it is normalized onto the template's coordinate system using nonlinear image transforms.^17^ Automated analysis tools are available for template‐based methods. However, cumulative errors introduced at each co‐registration step in this method are known to distort the reported positions of anatomical structures.

Accurate electrode localization not only serves clinicians on an individual patient level, but also provides researchers an endpoint measure for use in clinical trials. As datasets and multicenter collaborations continue to grow, the subjectivity of direct visualization is a confound.^18^ A standardized and more objective approach to electrode localization could allow for greater repeatability and consistent reporting across centers. Moreover, it could enable seamless pooling of data for studies with large sample sizes. The errors introduced and relative accuracy of alternative methods for the determination of DBS lead placement have not been systematically investigated and compared against the current standard and each other.

Here, we compare four methods of targeting the STN: direct visualization, RN‐based indirect targeting, MCP‐based indirect targeting, and automated template‐based targeting. We use the Euclidean distance from each target to the closest DBS electrode as a metric to investigate variability in anatomical targeting for electrode localization.

## METHODS

### Patient selection

People with PD were selected for STN DBS due to motor fluctuations and/or drug‐refractory tremor. One hundred and sixteen people with PD who received STN DBS consecutively between November 2013 and September 2017 were identified from a database. Data from revision surgeries were not included. The data were aggregated from a single neurosurgical team (WT, KB), operating across multiple centers in Melbourne, Australia: St. Vincent's, Austin, and Cabrini Hospitals.

This project was approved by the St. Vincent's Hospital Human Research Ethics Council (HREC/17/SCHM/81). A consent waiver was provided by the ethics committee for retrospective data collection where data obtained were deidentified at the point of collection. This dataset has been used previously to explore factors that may influence DBS electrode surgical placement errors.^19^

### Surgical procedure

**FIGURE 1:**
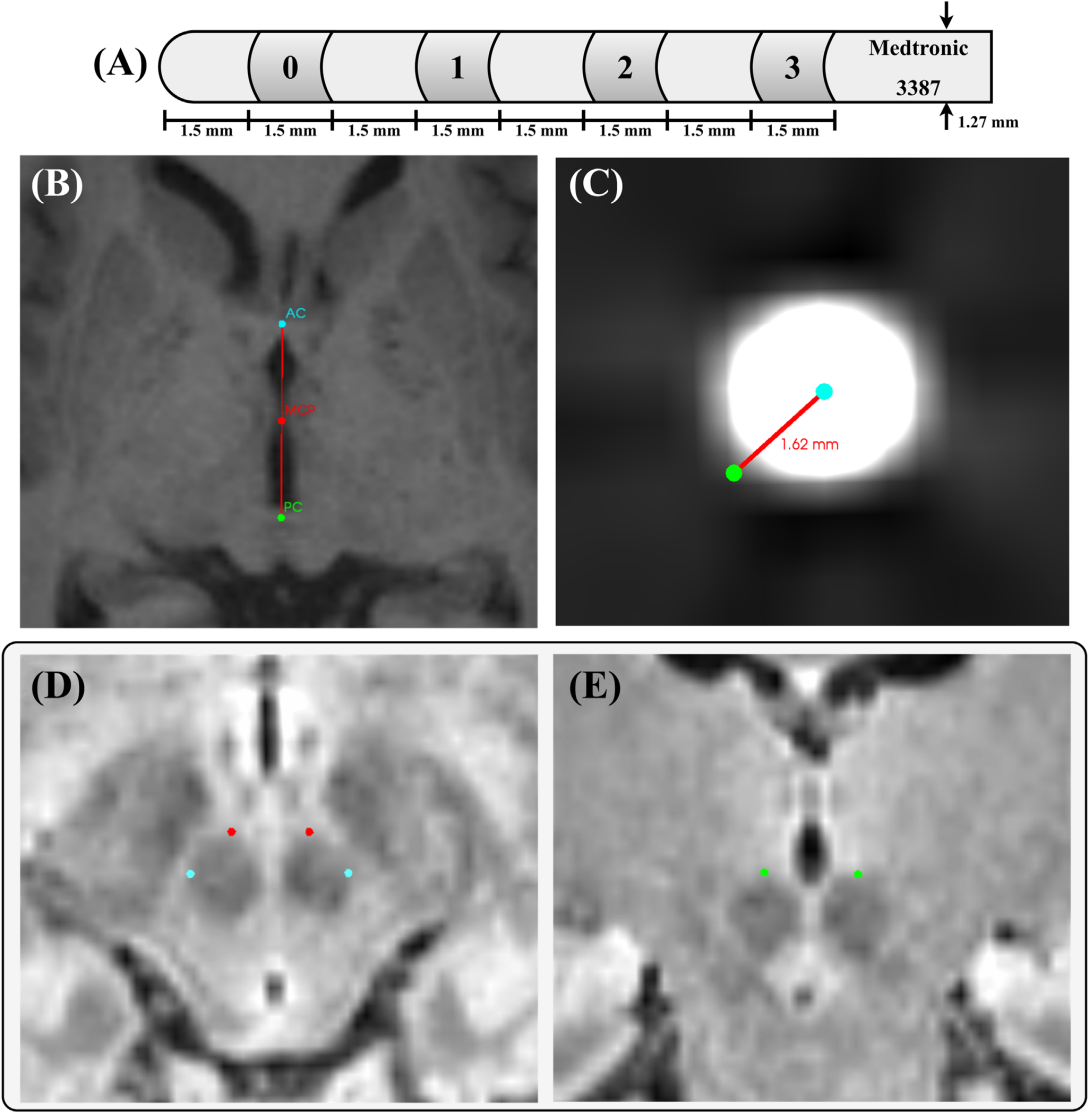
Deep brain stimulation lead diagram and anatomical markup examples. (A) Diagram of the deep brain stimulation leads utilized in this analysis: Medtronic 3387 leads with 1.5 mm tier spacing (four electrodes per lead). (B) The anterior commissure (AC) (cyan) and posterior commissure (PC) (green) and the mid‐commissural point (MCP) (red). The MCP is the midpoint of the line between the AC and PC. (C) The Euclidean distance (red line) that denotes the electrode placement error from the nominated target (green) for each method to the closest electrode (cyan; the midpoint of the CT artifact). (D) Axial fluid‐attenuated inversion recovery (FLAIR) MRI of the most lateral (cyan) and anterior (red) borders of the red nucleus. (E) Coronal FLAIR MRI of the most superior (green) borders of the red nucleus.

MRI at 3 Tesla (fluid‐attenuated inversion recovery [FLAIR] and T1 sequences) was acquired preoperatively in the axial plane (Table 1). For 13 cases, T1 images were not available. Bilateral implantation was performed with the patient awake using Medtronic 3387 DBS leads (four ring electrodes with 1.5 mm spacing) (Figure 1A). Surgical planning was performed by the neurologist (WT) on a Stealth Surgical Navigation System (Medtronic, Dublin, Ireland). Planning aimed to achieve a trajectory passing through the dorsal STN before reaching a termination point in the ventral STN midway between the medial‐lateral extent of the STN at the Bejjani line. Implantation was assisted by single‐ and multiunit recordings using the LeadPoint System (Medtronic) captured by microelectrodes (FHC, Bowdoin, Maine, USA). Postoperative CT imaging was acquired in the axial plane (Table 1) within 24 hours after surgery.

**TABLE 1:** Data collection summary.

T1‐weighted MRI parameters									
Site	Platform	Repetition time (ms)	Echo time (ms)	Inversion time (ms)	Matrix size	Number of slices	Slice thickness (mm)	In‐plane resolution (mm)	Field‐of‐view (mm)
Austin	Siemens Skyra	1900	2.43	900	256 × 256	(128, 256)	(0.137, 1.5)	1 × 1	(192 × 256, 512 × 512)
Cabrini	Siemens Prisma Fit	1900	2.28	900	256 × 224				
StVs	Siemens Magnetom	1900	2.52	900	256 × 246				

T2‐weighted MRI parameters									
Site	Platform	Repetition time (ms)	Echo time (ms)	Inversion time (ms)	Matrix size	Number of slices	Slice thickness (mm)	In‐plane resolution (mm)	Field‐of ‐view (mm)
Austin	Siemens Skyra	6000	388	2100	256 × 256	(128, 240)	(0.137, 1.5)	(0.087 × 0.186, 1 × 1)	(45 × 95, 224 × 256)
Cabrini	Siemens Prima Fit	5000	390	1800	256 × 256				
StVs	Siemens Magnetom	6000	388	2100	256 × 258				

CT parameters									
Site	Platform	Pitch factor	Tube voltage (kV)	Exposure current (mA)	Exposure time (ms)	Number of slices	Slice thickness (mm)	In‐plane resolution (mm)	Field‐of‐view (mm)
Austin	Toshiba Aquilion Prime SP	0.625	120	250	400	(136, 717)	(0.295, 1.25)	(0.391 × 0.391, 0.625 × 0.625)	(200 × 200, 320 × 320)
Cabrini	GE LightSpeed VCT	0.521	120	200	1095				
StVs	Siemens Somatom Definition Flash	0.55	120	212	1000				

*Note*: A summary of the neuroimaging data collection parameters for T1‐weighted MRI, T2‐weighted MRI, and CT imaging modalities used in this study. Data were collected across multiple centers in Melbourne, Australia: Austin, Cabrini, and St. Vincent's (StVs) hospitals. Cells containing comma separated values within brackets should be interpreted as (minimum, maximum).

### Manual imaging analysis

To localize the anatomical location of the implanted electrodes, manual processing was completed in 3D Slicer. First, preoperative MRI was transformed such that the coordinate axis aligned to the anterior commissure (AC), posterior commissure (PC), and the sagittal plane (Figure 1B). Second, the BRAINS module in 3D Slicer was used to co‐register postoperative CT (moving image) with preoperative FLAIR MRI (fixed image). Rigid+Scale+Skew (10 degrees of freedom) followed by Affine (12 degrees of freedom) transformations were used with linear interpolation and a sample percentage of 0.01. The Mattes Mutual Information cost metric was used to achieve maximal image overlap. All markups of necessary anatomical structures were performed on preoperative FLAIR MRI independently by two neurosurgeons (KB and PP) blinded to electrode location. The centroid coordinates for each of four electrode artifacts along the lead were marked independently on postoperative CT by a researcher (TP) (Figure 1C). The direct visualization, RN‐based indirect, and MCP‐based indirect methods utilized this manual process.

#### Direct visualization

A point was visually identified and manually marked in every hemisphere on preoperative MRI nominating an ideal location to apply DBS. This point was placed within the central area of the dorsal STN, 2 mm below the superior extent of the RN. This location was selected based on converging evidence suggesting this subregion to be the best location on average to apply STN DBS.^5^, ^8^

#### Red nucleus‐based indirect method

The borders of the RN were visually identified and manually marked as the most lateral (axial plane), anterior (axial plane), and superior (coronal plane) extents on preoperative MRI in all hemispheres (Figure 1D/E). The target was defined by reported offsets from these RN border points.^11^ This target was placed at the RN anterior border, 3 mm lateral of the RN lateral border, and 2 mm inferior of the RN superior border.

#### Mid‐commissural point‐based indirect method

The AC and PC were manually marked and the MCP was derived in all individuals, where the MCP is the midpoint of the line between the AC and PC (Figure 1B). The target was defined based on offsets: 12.02 mm lateral, 1.52 mm posterior, and 1.91 mm inferior to the MCP. This target originated from a systematic review that identified the average position of active electrodes in 342 leads targeting the STN for PD, representing an average ideal stimulation location relative to the MCP.^1^

### Automated template‐based method

A fully automated method was developed for this analysis by integrating multiple open‐source software packages into a neuroimaging analysis pipeline using Python (v3.8.10).^20^ The pipeline was designed to accept pre‐ and postoperative Digital Imaging and Communications in Medicine (DICOM) imaging and automate all necessary processing steps as well as apply an ideal target in template coordinate space. The main functions of the pipeline are as follows:

1. Convert neuroimaging data format from DICOM to Neuroimaging Informatics Technology Initiative (NIfTI) using dcm2niix (v1.0.201909902) as the algorithms employed in the following steps require NIfTI data.^21^
2. Crop image volumes to remove dark borders beyond the scalp using a fixed intensity threshold with the Nilearn Python library (v0.7.1).^22^
3. Perform AC‐PC alignment of preoperative MRI using the acpc‐detect algorithm (v2.0).^23^ This stage determines the positions of the AC, PC, and the midsagittal plane, before transforming the images to conform to a standard orientation (Figure 1B).
4. Co‐register postoperative CT with MRI using the BRAINSFit algorithm (v4.11).^24^ This stage aligns the CT with the MRI such that maximal overlap is achieved and coordinate systems are aligned (same parameters as the manual co‐registration).
5. Localize electrodes using the PaCER (v1.1.0) algorithm ported to Python.^25^ In this stage, the electrode artifacts along the lead in the CT images are identified and the centroid coordinates are detected.
6. Normalize imaging onto the Montreal Neurological Institute (MNI) 152 nonlinear 2009b asymmetric template (0.5 × 0.5 × 0.5 mm resolution) using ANTsPy (v0.2.2).^14^, ^15^ This stage transforms the individual's imaging onto a standard coordinate system by “fitting” the imaging to the template using a nonlinear deformation.^26^
7. Convert electrode coordinates determined by PaCER to MNI‐space. In this step, the normalization transformation applied to the images in the previous stage is also applied to the electrode coordinates.
8. Denote the target using MNI coordinates *x* = ±12.58, *y* = −13.41, and *z* = −5.87. This is a reported average target transformed to MNI‐space.^1^, ^16^

### Statistical methods

Interrater reproducibility analysis of markups made by the two independent neurosurgeons was conducted to assess the reliability of markups used in the three manual methods. Reproducibility analysis for the markup of both the STN and the RN was performed. The Euclidean distance between the target and the image origin (0, 0, 0) for markups by both neurosurgeons was calculated in all hemispheres. The intraclass correlation coefficient (ICC) using the ICC (2, 1) model was calculated for both the STN and the RN. All further analysis utilized markups defined by KB only.

To compare the relative accuracy of each method, the Euclidean distance from the nominated target to the closest actual electrode (electrode placement error) was calculated (Figure 1C). This was completed for all DBS leads using all four methods (direct visualization, RN‐based indirect, MCP‐based indirect, and automated template‐based method) separately. Only the closest electrode was utilized in this analysis as it held the most relevance in its likelihood to be chosen for stimulation.

To assess the concordance of the alternative methods with direct visualization as well as each other, the difference between the electrode placement errors was calculated pairwise in all hemispheres and plotted in histograms. This resulted in six comparisons. The greater the similarity between methods, the higher the density around 0 mm difference in electrode placement error. Similarity between methods was quantified using the interquartile range, along with the number of electrodes within 0.5, 1, and 2 mm difference in electrode placement error.

Shapiro‐Wilk tests for normality were completed for all comparisons. A one‐way analysis of variance (ANOVA) was performed for all six comparisons to reveal if the mean difference of any of the comparisons was significantly different from the rest. If a statistically significant difference was found, t‐tests were performed for each comparison. Where Shapiro‐Wilk tests reported nonnormality for any of the comparisons, nonparametric versions of one‐way ANOVA and *t*‐tests (Kruskal‐Wallis *H*‐test and Wilcoxon signed‐rank tests) were employed instead.

Separately, six confusion matrices were tabulated comparing the closest electrodes selected by each method to reveal any systematic difference in selection. In a confusion matrix, two methods are compared. Each row and column represent an electrode, and the individual cells present the number of instances where both methods selected the corresponding electrodes as closest to their respective targets. If each method was identical to each other, then the closest electrodes will appear along the diagonal of the confusion matrix. Statistical differences were deemed significant if *p* < .05. Bonferroni correction was applied to control for the inflation of error due to multiple comparisons. All data processing and statistical analysis were performed in Python (v3.8.10)^20^ using the Pandas (v1.3.1),^27^ SciPy (v1.5.4),^28^ and Pingouin (v0.5.3)^29^ packages.

## RESULTS

From the initial dataset of 116 participants, two were excluded due1, 2 to incomplete imaging data, and one was excluded due to low CT resolution (slice thickness >1.5 mm) that was unsuitable for the electrode localization algorithm (PaCER) utilized in the automated template‐based method.^25^ Consequently, data from a total of 113 participants (226 STNs) were retrospectively analyzed. The AC‐PC alignment algorithm (acpc‐detect) used in the automated template‐based method failed in 19 cases. This was due to either acpc‐detect encountering incompatible imaging data types (anything that is not a short or unsigned short) or unsatisfactory alignment upon visual inspection. For these cases, AC‐PC alignment was completed manually.

**FIGURE 2:**
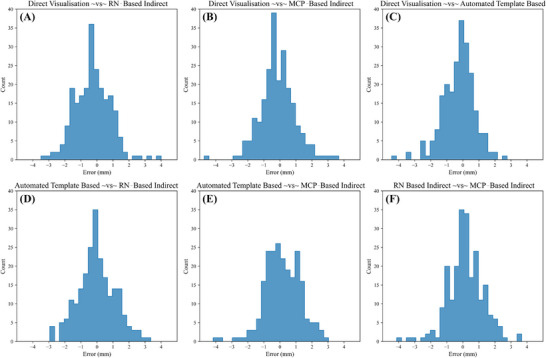
Distributions of the differences in electrode placement error between methods. Histograms of the differences in electrode placement error between each of the four targeting methods for electrode localization, resulting in six comparisons. The four methods compared were direct visualization, red nucleus (RN)‐based indirect method, mid‐commissural point (MCP)‐based indirect method, and an automated template‐based method.

The ICC for the STN markups was .981 (95% confidence interval [CI]: .97‐.99), while the ICC for the RN markups was .991 (95% CI: .99‐.99), indicating excellent reliability in both (*p* < .001). Figure 2 and Table 2 document the comparative analysis results. Interquartile ranges for all comparisons ranged between 1.18 and 1.56 mm. The direct visualization and automated template‐based methods yielded the greatest number of electrodes within 0.5 mm difference (104 electrodes). The two methods also yielded the greatest similarity in closest electrode selection (143 electrodes).

**TABLE 2:** Comparative analysis summary.

Comparison	IQR (mm)	Within 0.5 mm difference	Within 1 mm difference	Within 2 mm difference
DV vs. RN	1.47	85	142	212
DV vs. MCP	1.18	92	159	211
DV vs. Auto	1.24	104	158	213
Auto vs. RN	1.38	89	138	209
Auto vs. MCP	1.56	77	149	208
RN vs. MCP	1.26	93	154	210

*Note*: A summary of the six comparisons between the four targeting methods for subthalamic nucleus deep brain stimulation (DBS). The four methods compared were direct visualization (DV), red nucleus‐based indirect (RN), mid‐commissural point‐based indirect (MCP), and automated template‐based (Auto) methods. Comparative analysis was performed by calculating the differences between electrode placement error (Euclidean distance between the anatomical target defined by each method and the closest actual DBS electrode) of each method. Interquartile ranges (IQRs) were calculated from the distributions of the differences in electrode placement error. The cumulative number of electrodes within 0.5, 1, and 2 mm difference in electrode placement error out of a possible 226 is also presented.

Only two comparisons had normal distributions (*W* > .989, *p* > .05): direct visualization versus RN‐based indirect, and automated template‐based versus MCP‐based indirect comparisons. Thus, nonparametric statistics were used for all post hoc tests. A Kruskal‐Wallis *H*‐test revealed that there was a statistically significant difference in the median between at least two groups (*H*(5) = 41.052, *p* < .001). Post hoc Wilcoxon signed‐rank tests after Bonferroni correction reported a statistically significant difference in the distributions of two comparisons: direct visualization versus RN‐based indirect, and direct visualization versus automated template‐based methods (*T* < 9215, *p* < .001). For the remaining four comparisons, the distributions did not differ (*T* > 10,282, *p* > .05).

**FIGURE 3:**
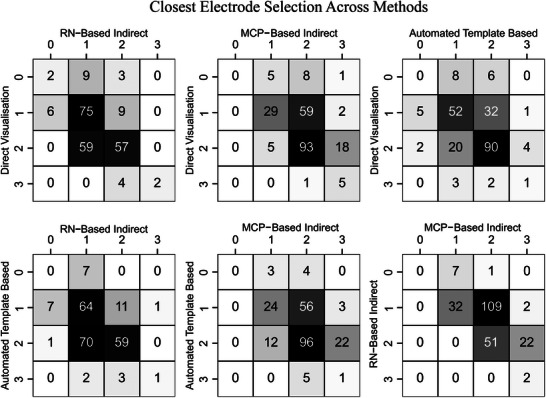
Closest electrode selection confusion matrices. Confusion matrices comparing the closest electrodes selected by each of the four targeting methods against each other. The four methods compared were direct visualization, red nucleus (RN)‐based indirect method, mid‐commissural point (MCP)‐based indirect method, and an automated template‐based method. Numbers on the *X*‐ and *Y*‐axis correspond to the individual electrodes along the deep brain stimulation lead (Figure 1A). If each method was identical in closest electrode selection, then nonzero values would be confined along the diagonal of the confusion matrices.

Tabulated confusion matrices (Figure 3) comparing the closest electrodes selected by each method with each other displayed a variation in selection, primarily between neighboring electrode tiers. Closest electrode selection was most agreeable between direct visualization and the automated template‐based method (143 electrodes) against the other comparisons (85–136 electrodes).

## DISCUSSION

Here, in a final cohort of 113 participants (226 STNs), we retrospectively compared four different methods of targeting the STN for electrode localization. The methods included direct visualization, RN‐based indirect, MCP‐based indirect, and an automated template‐based method. Comparative analysis revealed similarity in interquartile ranges for the differences in electrode placement error across all methods (Table 2; Figure 2). These results highlight that all methods are similar in their relative accuracy, despite having significant technical differences in their application. This, however, provides evidence that certain methods can be employed in situations where they may be more suitable than others without significantly affecting targeting accuracy. Direct visualization will likely remain a key aspect of targeting the STN in the clinical environment as it enables greater accuracy on the individual level than any other technique. Indirect methods hold greatest relevance for analyses requiring electrode localization in small datasets where experts may not be available. Lastly, automated analysis pipelines, such as the one developed for this study, will be more applicable in large‐scale datasets where manual marking is impractical.

Interquartile ranges for all six comparisons differed within a maximum of 0.38 mm, indicating all four methods yield similar overall results and could be used interchangeably. Wilcoxon signed‐rank tests showed a statistical difference in the distributions of two comparisons (direct visualization vs. RN‐based indirect, and direct visualization vs. automated template‐based methods), with a shift in the median of the differences of −0.29 and −0.17 mm from zero, respectively. We believe that the large sample size may have increased the sensitivity of the tests to small shifts in the distributions of the methods compared. We argue that such small shifts in the distributions are not practically significant, being less than the image resolutions produced by standard MRI protocols.

The confusion matrices (Figure 3) show significant discrepancies in selection of the closest electrode between the methods tested. In studies that evaluate new implantation techniques and postoperative programing that use electrode localization, the Euclidean distance to the closest electrode is often used as an outcome measure.^30^ This analysis reveals that closest electrode selection varies significantly across methods, particularly between neighboring electrodes. For the dataset utilized, surgical planning aimed to position the two middle electrodes within the STN, while the tip electrode (labeled zero in Figure 1A) was positioned within the substantia nigra pars reticulata. This likely explains why closest electrode selection is concentrated across the two middle electrode tiers. This study used Medtronic 3387 DBS leads with a spacing of 1.5 mm between tiers, while many centers use leads with smaller between‐electrode spacing of 0.5 mm. We expect that the observed discordance in the closest electrode selection in this analysis may be even greater in leads with tighter spacing.

The similar relative accuracy of the automated template‐based method to the other methods in this analysis is notable due to its utilization of an end‐to‐end software pipeline that performed all necessary processing. Unlike the other methods in this analysis, this method can be considered fully objective and less labor‐intensive. These characteristics allow for a more resource‐efficient and potentially more appropriate method than subjective techniques in certain scenarios. Standardized automated systems could also facilitate multicenter analysis of large‐scale datasets that evaluate electrode localization to identify spatial correlates of treatment outcomes and other similar investigations.^18^ It is important to note, however, that some components of the current automated analysis pipelines can be unstable, as seen in this analysis, where the AC‐PC transformation algorithm failed in 19 cases. As a result, all outputs from automated analysis should be manually reviewed.

The primary limitation of this study is the use of a single expert for direct visualization and markup of structures for both RN‐ and MCP‐based indirect methods, which may introduce subjectivity and variability that affects the results and their reproducibility. This limitation also extends to the manual merging of preoperative MRI and postoperative CT that may influence electrode placement accuracy. Direct visualization relies on the clinician's expertise and understanding of anatomy, facilitating unwanted variation between experts.^1^ To minimize this limitation as a confound, interrater reproducibility analysis between two neurosurgeons revealed a high level of agreement (ICC > .981) for both direct visualization of the STN and identification of the RN borders. While this does not mitigate entirely the challenge of subjectivity when performing electrode localization, it does assist in the generalizability of the results presented.

Indirect methods aim to reduce subjectivity by using markups of structures that are more invariant across participants and more readily visualized on MRI. However, the use of population‐based probabilistic offsets, interindividual variances in the spatial relationship between nuclei, and variation in how reference points are marked can introduce error comparable to direct visualization.^11^ Moreover, indirect methods were originally developed to work around the limitations of neuroimaging of the time. Since their implementation, imaging resolutions have greatly improved, making direct visualization of the STN more favorable in the clinical environment. Manual markups remain a significant challenge for electrode localization and are an argument for transitioning toward more objective and reproducible techniques.^1^

The automated template‐based method in this analysis also suffers from the limitation of probabilistic targets that may be inaccurate at the individual level.^10^ Conversion of participant imaging to MNI‐space aims to minimize this variability, but the nonlinear deformation process is known to distort anatomy introducing error. Additionally, the algorithms used in the automated method may produce suboptimal outputs or fail, requiring manual intervention, as seen in this study.

A further limitation important to note is potential variation as a result of poor merging of the CT and MRI. Comparisons between the manual methods in this analysis are not affected as each technique used the same manual merges. However, the automated template‐based method utilized an automated merging algorithm (BRAINSFit) that may introduce variation in electrode placement error, affecting the results in comparisons containing the automated template‐based method.

This analysis highlights the ongoing challenge of accurately applying an ideal anatomical target for STN DBS on an individual level. While probabilistic targets provide accuracy at the group level, they may not be accurate for individuals. Advances in imaging, probabilistic targets, and processing techniques have made significant progress in the field, but several challenges remain. Current methods show similar levels of relative error, regardless of the techniques used to target the STN. The differing protocols and technical aspects of each method, however, have the implication that one may be more practical depending on the clinical or research application at hand. We speculate these findings may be transferrable to other DBS targets. While automated systems are currently still comparably inaccurate and sometimes unstable, we expect them to become increasingly integrated into neuroimaging research for DBS as further advances are made in connectomics and other avenues of computational neuroscience.

## CONFLICT OF INTEREST STATEMENT

TT, PP, and NA report no conflicts of interest. HM, WT, and KB are co‐founders and hold shares and options in DBS Technologies Pty Ltd that plans to commercialize the use of neuronal signals to improve DBS. They are also named inventors on related patents, which are assigned to DBS Technologies Pty Ltd. SSX holds options in DBS Technologies Pty Ltd. TP receives consulting fees, holds options, and is a named inventor on related patents assigned to DBS Technologies Pty Ltd.
